# Clinical management, pathogen spectrum and outcomes in patients with pyogenic liver abscess in a German tertiary-care hospital

**DOI:** 10.1038/s41598-024-63819-w

**Published:** 2024-06-05

**Authors:** Sebastian Wendt, Miroslav Bačák, David Petroff, Norman Lippmann, Valentin Blank, Daniel Seehofer, Lisa Zimmermann, Christoph Lübbert, Thomas Karlas

**Affiliations:** 1 Hospital Hygiene Staff Unit, University Hospital Halle (Saale), Halle (Saale), Germany; 2 Division of Infectious Diseases and Tropical Medicine, Department of Medicine I, Leipzig University Medical Center, Leipzig, Germany; 3 Clinical Trial Center Leipzig, University of Leipzig, Leipzig, Germany; 4 Interdisciplinary Center for Infectious Diseases (ZINF), Leipzig University Medical Center, Leipzig, Germany; 5 Institute for Medical Microbiology and Virology, Leipzig University Medical Center, Leipzig, Germany; 6 Division of Gastroenterology, Department of Medicine II, Leipzig University Medical Center, Liebigstraße 20, 04103 Leipzig, Germany; 7 Division of Interdisciplinary Ultrasound, Department of Medicine I, University Hospital Halle (Saale), Halle (Saale), Germany; 8 Department of Visceral, Transplantation, Thoracic and Vascular Surgery, Leipzig University Medical Center, Leipzig, Germany

**Keywords:** Clinical microbiology, Gastroenterology, Infectious diseases

## Abstract

Pyogenic liver abscesses (PLA) are life-threatening disorders and require immediate treatment, but structured evidence is sparse and treatment guidelines are not established. In a retrospective observational study of 221 adult PLA patients (mean age 63 years, 63% men) treated between 2013 and 2019 at the Leipzig University Medical Center, we characterized pathogen spectrum, clinical management and outcomes. Biliary malignancies (33%), cholelithiasis (23%) and ischemic biliary tract disease (16%) were most common causes of PLA. Comorbidities included malignancies (40%) and diabetes mellitus (35%). Abdominal ultrasound was the preferred initial imaging modality (58%). Enterobacterales (58%), enterococci (42%) and streptococci (18%) were identified as most frequent pathogens. 97% of patients were treated with antibiotics and 75% of patients underwent an invasive treatment procedure. The 30-day mortality was almost identical in patients with and without underlying malignancy (14.6% vs. 14.4%, p = 0.96), while the one-year outcome differed significantly (58.4% vs. 29.6%, p < 0.001). Positive blood cultures (OR 4.78, 95% CI 1.39 to 22.5, p = 0.023) and detection of Enterobacterales (OR 3.55, 95% CI 1.40 to 9.97, p = 0.010) were associated with increased 30-day-mortality. We conclude that ultrasound, extensive microbiologic diagnosis, adequate anti-infective therapy and early intervention are crucial for the management of PLA.

## Introduction

Liver abscesses (LA) represent a rare but potentially life-threatening abdominal condition that requires immediate diagnosis and therapy. Due to differences in the prevalence of pathogens and hygiene standards, pathogen etiology varies by geographic location^1^. In Asia, where amoebic liver abscesses (ALA) predominate and hypervirulent invasive *Klebsiella pneumoniae* strains as well as *Burkholderia pseudomallei* are important etiological agents, the overall LA incidence rates reach 15 to 18 per 100,000 person-years^1–13^. Incidence rates in Europe and North America range from 1 to 7 per 100,000 person-years, with pyogenic LA (PLA) accounting for the vast majority (more than 80%) of LA cases^14–19^. Although hypervirulent *Klebsiella* strains are emerging pathogens in Western countries, they still play a minor role in the etiology of LA in this part of the world^20,21^. However, there is evidence that LA is an infectious disease with a markedly increasing incidence in both Western countries and Asia^4,15,16,18,19^.

While ALA can usually be treated effectively with metronidazole therapy, PLA is a syndrome and the consequence of numerous underlying diseases in very heterogeneous patient populations^22^. PLA represents a serious complication with relevant morbidity, especially in cancer patients and liver transplant recipients^14–19^. Unfortunately, the available evidence on the clinical management of PLA is largely based on case series, retrospective observational studies, expert opinions, and few randomized controlled trials, mainly from non-European countries^14,23–30^. National clinical guidelines are not yet available.

Our study aims to characterize the diagnostic and therapeutic management, the pathogen spectrum and the outcome in PLA patients using data from a large German tertiary-care hospital over a 7-year period.

## Methods

### Ethics and data privacy

The approval of the local ethics committee was obtained in advance (University of Leipzig, register no. 170/20-ek). Due to the retrospective observational design, informed consent was waived. The study protocol conforms to the ethical guidelines of the 1975 Declaration of Helsinki as reflected in a priori approval by the institution's human research committee.

### Study design and setting

This retrospective, observational single-center study was conducted at the departments of internal medicine and surgery of the Leipzig University Medical Center (LUMC), which is a 1451-bed tertiary-care facility with a well-developed liver transplant center. The inclusion period was chosen to be from January 1, 2013 through December 31, 2019. Inclusion criteria comprised adult (≥ 18 years) patients with confirmed main or secondary diagnosis of LA. Cases with contradictory or misclassified diagnoses as well as outpatients were excluded.

### Endpoints

Endpoints were the frequency of LA by year, etiology (hematogenous, biliary, parasitic, unclear), and the outcomes defined by 30-day and one-year all-cause mortalities. Because patients in this retrospective study could not be contacted due to privacy laws, data on mortality that were not available in the electronic records had to be obtained by searching public records, and missing data were unavoidable.

Further endpoints included frequency of symptoms, comorbidities, medications prior to diagnosis, initial and additional imaging, morphology and location of abscess(es), laboratory findings, pathogen spectrum, and therapeutic approach.

### Data sources and case reviews

A structured data warehouse search was performed through the hospital information system (HIS) *i.s.h.med*® (Cerner Health Services, Berlin, Germany) and the picture archiving and communication system (PACS) *ViewPoint*® 5.0 (GE HealthCare, Solingen, Germany) using ICD-10 codes and predefined text terms.

Cases were identified using standard codes of the 10^th^ version of the international statistical classification of diseases and related health problems (ICD-10): K75.0 (“liver abscess”), K77.0* (“liver disease associated with infectious and parasitic diseases”), and A06.4 + (“amebic liver abscess”). In addition, a PACS search was performed using the free-text term *abs* which potentially refers to LA (German: “Leberabszess”) in sonographic and endoscopic examinations.

Duplicate cases were removed, and potentially relevant patients were grouped for further analysis if inclusion criteria were met. Subsequently, medical records and all medical findings (imaging, laboratory values, microbiological findings, treatment protocols) of patients with potential LA were reviewed according to the registered study protocol.

An intervention (e.g. puncture, drainage, surgery) was defined as *primary* therapy if it was initiated within 3 days after diagnosis irrespective of an earlier initiation of targeted antibiotic therapy.

Baseline characteristics data refer to the day of diagnosis ± 3 days if values were not available for that day. For external diagnoses, baseline data refer to either the day on which the diagnosis was made or, if no values were available, the day of admission to LUMC.

### Abscess material and microbiological analyses

Local abscess cultures were obtained primarily by aspiration or drainage, and in a few cases by endoscopic retrograde cholangiopancreaticography (ERCP) or intraoperatively.

Microbiological examinations were performed as part of routine diagnostics by the hospital's microbiology laboratory. Puncture fluids, aspirates, biopsies, and tissues were cultured using standard microbiological methods, followed by standard antimicrobial susceptibility testing (AST). Only initial cultures were considered for microbiological diagnoses. ALA were diagnosed serologically (IgG antibody detection for *Entamoeba histolytica* in ELISA, Novagnost^®^, NovaTec/Siemens, Dietzenbach, Germany) in combination with imaging and the absence of other conclusive causes of LA.

### Statistical analysis

We used descriptive statistical methods and the Kaplan–Meier estimator to approximate survival functions while accounting for censoring^31^. At 30 days, contingency tables with a chi-squared test without Yate’s correction was used and odds ratios (OR) were determined by multivariable logistic regression. Two sets of covariates were considered here, namely: age, positive blood culture(s), malignancy, obesity, diabetes, liver transplantation, monomicrobial/polymicrobial infection in the first set, and age, sex, malignancy, obesity, diabetes, liver transplantation, anaerobic bacteria, enterococci, Enterobacterales, and fungi in the second set. Hazard ratios were obtained from a classic Cox model with covariates: age, malignancy, *Candida* species, Enterobacterales and Enterococci. The significance level for all statistical tests was set at 0.05 two-tailed. All statistical analyses were performed using R® 4.2.2 statistical software^32^.

### Reporting

Study results are presented in accordance with the *Strengthening the Reporting of Observational Studies in Epidemiology* (STROBE) statement^33^.

## Results

### Search results

**Figure 1:**
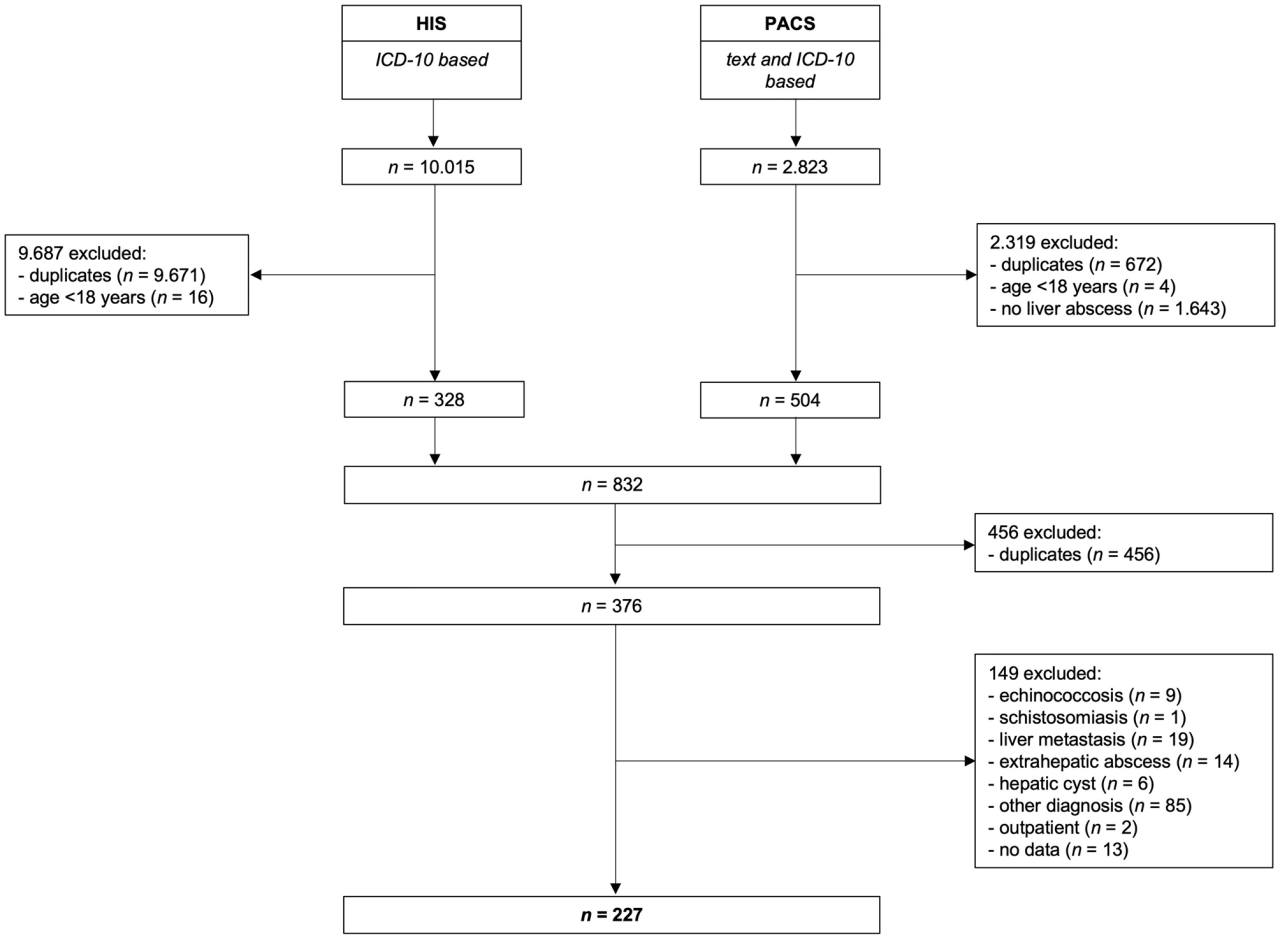
Overview of the search strategy and the patients included in the study (*HIS* hospital information system, *PACS* picture archiving and communication system, *ICD-10* 10th version of the international statistical classification of diseases and related health problems).

A total of 376 patients with potential LA were identified by the search terms (Fig. 1). After detailed review of the preselected cases, 227 patients met the inclusion criteria.

### Baseline characteristics

The baseline characteristics of 227 patients with LA are shown in detail in Table 1. The annual number of LA cases did not differ significantly within the study period (2013: n = 17, 2014: n = 28, 2015: n = 31, 2016: n = 40, 2017: n = 30, 2018: n = 35, 2019: n = 40, mean: 32 cases per year, range: 17–40).

**Table 1:** Baseline characteristics of the study population.

Baseline characteristics	ALA, 6 (2.6%)	PLA, 221 (97.4%)
Sex, male, *n* (%)	5 (83.3%)	140 (63.3%)
Age, median years (range)	47.5 (22–72)	63.0 (27–90)
Travel abroad within 1 year, *n* (%)	5 (83.3%)	*NA*
Medication prior to diagnosis		
Anti-infectives, *n* (%)	0 (0%)	8 (3.6%)
Anticoagulants, *n* (%)	0 (0%)	27 (12.2%)
Immunosuppressives, *n* (%)	0 (0%)	31 (14.0%)
Proton-pump inhibitor (PPI), *n* (%)	1 (16.7%)	120 (54.3%)
Other, *n* (%)	4 (66.7%)	154 (69.7%)
None, *n* (%)	1 (16.7%)	7 (3.2%)
Unknown, *n* (%)	0 (0%)	13 (5.9%)
Typical symptoms of LA, *n* (%)	5 (83.3%)	141 (63.8%)
Ascites, *n* (%)	0 (0%)	89 (40.3%)
Laboratory findings		
CRP, mg/L median (range)	74 (2–341)	151.5 (1–490)
WBC count, 10^9^/L, median (range)	13.6 (5.2–16.3)	12.5 (0.7–34.6)
PCT, ng/mL, median (range)	1.1 (1.1- 1.1)	3.6 (0.06–701)
HbA1c, %, median (range)	*NA*	5.2 (4.3–9.3)
ASAT, µkat/L, median (range)	0.52 (0.34–1.5)	0.84 (0.23–53)
ALAT, µkat/L, median (range)	0.72 (0.24–1.8)	0.75 (0.08–27.6)
GGT, µkat/L, median (range)	1.2 (0.26–2.15)	3.5 (0.07–36.3)
Bilirubin (total), µmol/L, median (range)	7.45 (3.2–16.6)	16 (2.5–335)
Bilirubin (direct/conjugated), µmol/L, median (range)	*NA*	39.4 (3–226)
GFR, mL/min/1.73 m^2^, median (range)	111 (82–134)	81 (2.4–149)
TPZ, %, median (range)	66 (47–99)	67 (8–116)
PT/INR, median (range)	1.5 (1.3–1.7)	1.3 (0.9–7.9)
< 1.5, *n* (%)	3 (50%)	159 (72%)
1.5–2, *n* (%)	2 (33%)	44 (20%)
> 2, *n* (%)	0 (0%)	2 (1%)
Abscess characteristics		
Solitary abscess, *n* (%)	6 (100%)	120 (54%)
Multiple abscesses, *n* (%)	0 (0%)	99 (45%)
Count unknown, *n* (%)	0 (0%)	2 (1%)
Largest diameter, mm, median (range)	65.5 (40–113)	50 (8–240)
Right lobe, *n* (%)	6 (100%)	133 (60%)
Left lobe, *n* (%)	0 (0%)	51 (23%)
Both lobes, *n* (%)	0 (0%)	12 (5.4%)
Unknown lobe(s), *n* (%)	0 (0%)	25 (11%)

*NA* not assessed.

PLA was diagnosed in 221 patients (median age 63 years, 63% male), whereas ALA was diagnosed in only 6 cases (median age 48 years, 83% male). Given the low incidence of ALA, we focused our analysis on PLA cases.

### Comorbidities of PLA patients

**Figure 2:**
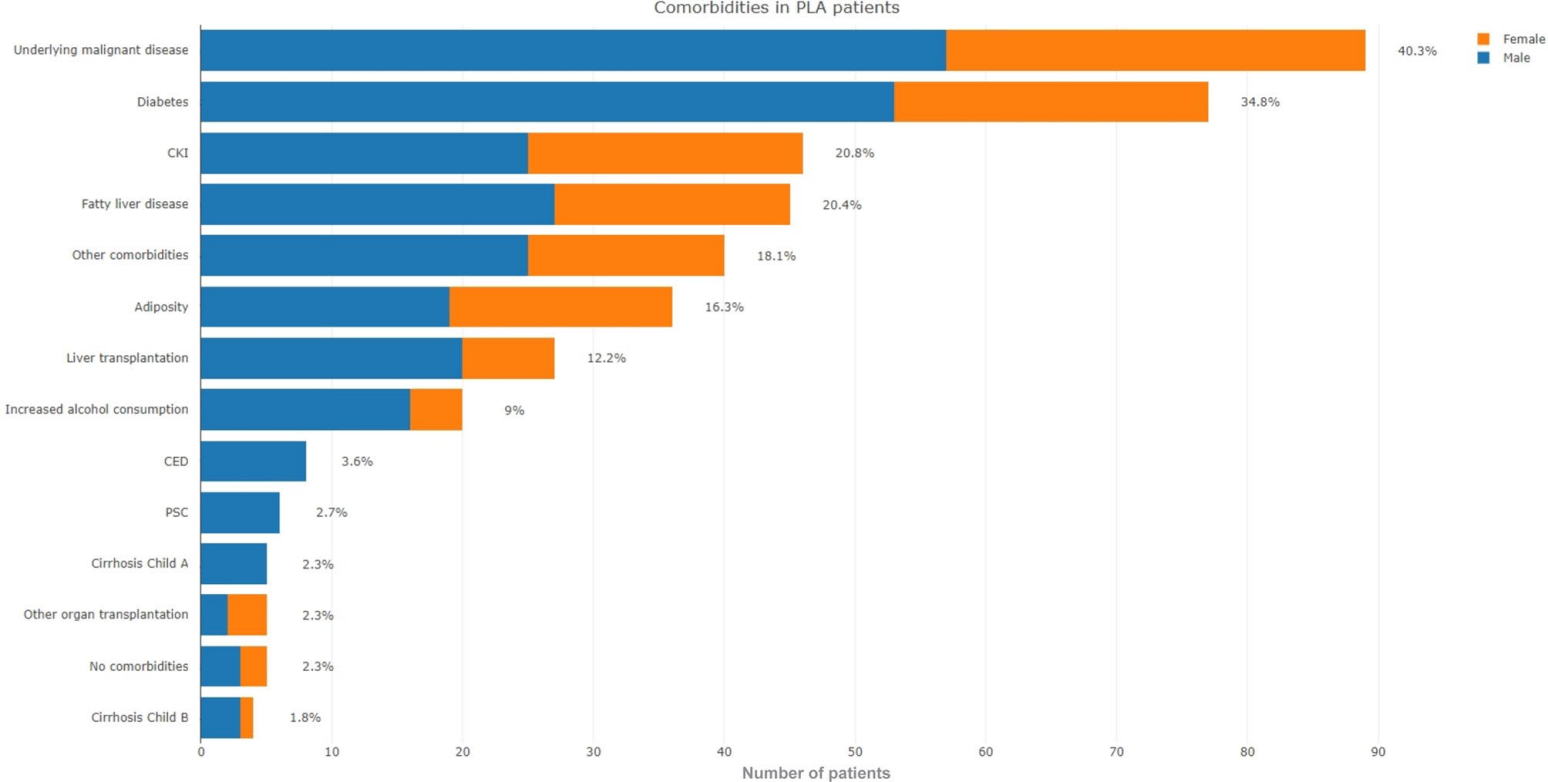
Comorbidities of patients with pyogenic liver abscess.

Most frequently, PLA patients had underlying malignancy (40%), diabetes mellitus (35%), chronic kidney disease (21%), or fatty liver disease (20%) (Fig. 2). Only 5 (2%) patients had no comorbidities.

### Diagnostic cascade

**Figure 3:**
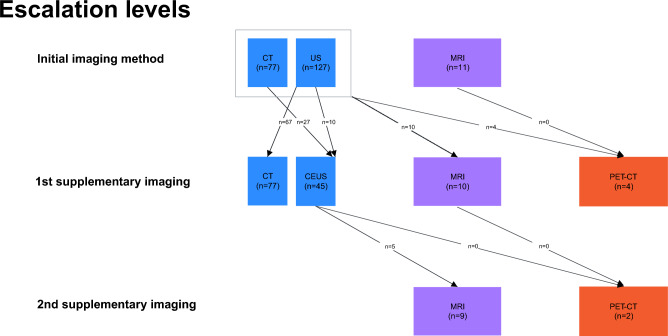
Diagnostic tree confirming diagnoses of pyogenic liver abscess in the study population (*CT* computed tomography, *US* ultrasound, *MRI* magnetic resonance imaging, *PET* positron emission tomography).

Ultrasound (US) was used as the initial imaging modality to diagnose PLA in 127 (58%) patients, computed tomography (CT) in 77 (35%), and magnetic resonance imaging (MRI) in 11 (5%) cases (Fig. 3).

In the remaining 6 cases, the diagnosis was made in 3 patients by endoscopic retrograde cholangiopancreatography (ERCP), intraoperatively in 2, and 1 patient was referred from an external hospital. A first supplemental imaging was performed in 59 (27%) patients, and a second supplemental imaging in 11 (5%) patients.

### Risk factors and etiology

The most common cause of PLA was malignant biliary disease (72 patients; 33%), followed by biliary cholelithiasis (51; 23%), and ischemic bile duct disease (36; 16%). Only 11 (5%) cases were considered to have a hematogenous etiology. In 51 (23%) cases (36 male; 4 with underlying malignancy), the cause of PLA remained unclear.

### Microbiological characteristics

Abscess punctates were obtained in 163/221 (74%) patients, and blood cultures in 173/221 (78%) patients. Both materials were processed in 130/221 (59%) patients. PLA cultures were positive in 143/163 (88%) patients, and blood cultures in 94/173 (54%) patients. Both culture types were positive in 66/130 (51%) cases, with conclusive pathogens in 46/66 (70%) of these cases (Supplementary Table S1 online). 103/171 (60%) PLA were defined as polymicrobial infections.

Enterobacterales (100; 58%), led by the species *Escherichia (E.) coli* (61; 36%) and *Klebsiella* spp. (43; 25%) were most frequently identified, followed by enterococci (71; 42%) and streptococci (31; 18%) (Supplementary Table S2 online). *Candida* species (29; 17%,) and anaerobes (24; 14%) were detected less frequently, followed by non-fermenters (13; 8%) and staphylococci (13; 8%).

Yearly summaries of the general resistance rates of Enterobacterales and enterococci were accessible for our hospital throughout the study period (supplementary Table S3 online). These data indicate relatively low resistance rates across antibiotic main classes and demonstrate a slight decrease in resistance rates for several strains over time. Notably, there were no significant resistance rates observed for fungal infections caused by *Candida albicans*.

### Therapeutic cascade

**Figure 4:**
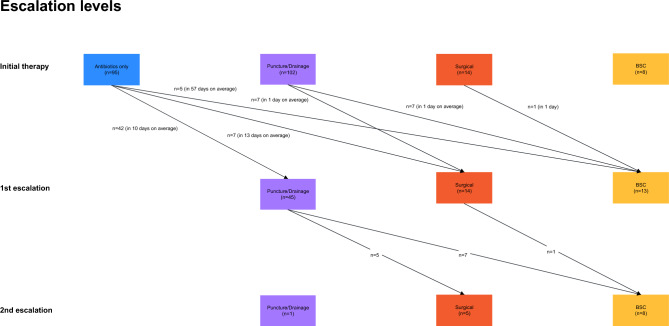
Therapeutic cascade in the treatment of patients with pyogenic liver abscess.

Almost all patients received antibiotic treatment (214; 97%). In addition, the initial therapeutic regimen included image-guided interventions (102; 46%) within the first 3 days, whereas 95 patients (43%) underwent a conservative procedure with antibiotic therapy only. Initial surgery (14; 6%) and immediate best supportive care (BSC) decisions (8; 4%) were less common (Fig. 4). The 30-day mortality in patients who received an initial antibiotic therapy was 9.5%, compared to 15.5% in those who received an invasive therapy (puncture, drainage, surgery). The mortality difference of 6.0% (95% CI  − 2.8% to 14.9%) was however not statistically significant (p = 0.19). A large proportion of patients with primary antibiotic therapy (42/95; 44%) received abscess puncture or drainage as first escalatory strategy after day 3 within a mean period of 10 days. In total, 166/221 (75%) of patients received a diagnostic or therapeutic procedure.

Few patients required further escalation of therapy and underwent surgery (5; 2%). 29 (13%) cases were eventually converted to BSC, and 22 (10%) died.

### Anti-infective therapy

Antibiotic therapy was continued for a median of 15 days (range 0–176), with a median time from diagnosis to first administration of 0 days (range 0–3). Broad-spectrum antibiotics including carbapenems (*n* = 96) and piperacillin/tazobactam (*n* = 92) were used most frequently, followed by metronidazole (*n* = 70), fluoroquinolones (*n* = 56), and cefotaxime (*n* = 49) (Supplementary Table S4 online).

### Survival analysis

All-cause mortality analyses were based on an observation period of 1 year after which data were censored.

**Figure 5:**
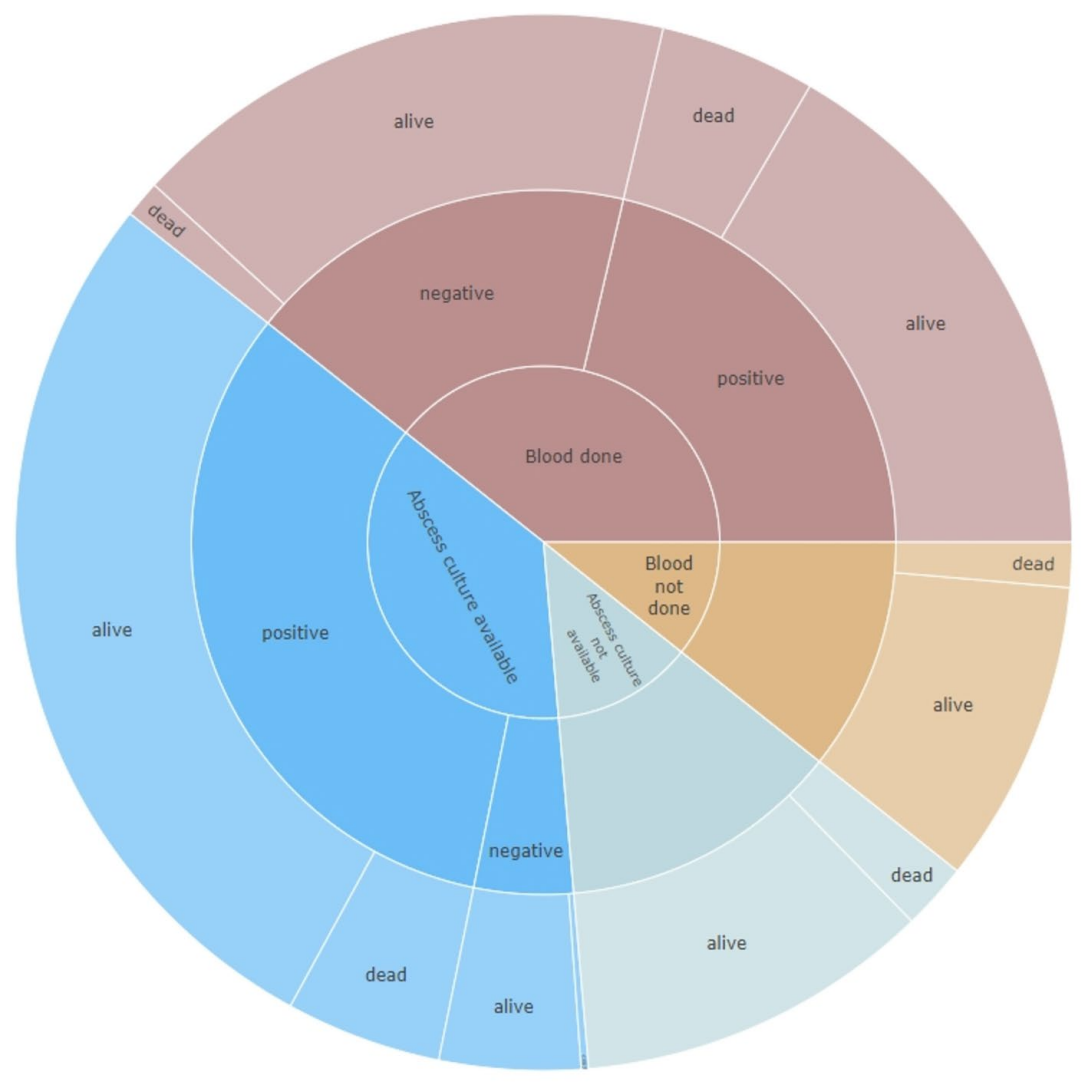
Sunburst chart showing all-cause survival ratios 30 days after diagnosis of pyogenic liver abscess as a function of blood and abscess culture results. When interpreting the diagram, it is important to note that a semicircle (in blue and red tones, respectively) in each case reflects the whole collective (100%) of PLA patients.

At 30 days after diagnosis, 32 patients were known to have died. Treating the others as alive shows that mortality did not differ between those with malignancies (13/89; 14.6%) and those without (19/132; 14.4%) for a difference of 0.2 percentage points (95% CI − 9.3 to 9.7 percentage points, p = 0.96). The association between 30-day mortality and pathogen detection in blood or PLA cultures is shown in Fig. 5. Patients with positive blood cultures had an increased risk of death (OR 4.78, 95% CI 1.39 to 22.5, p = 0.023), though 103 patients had missing data and were not accounted for in the model. A sensitivity analysis including all patients for whom blood culture data were available led to similar results. The risk of death in PLA patients was also increased when Enterobacterales were detected (OR 3.55, 95% CI 1.40 to 9.97; p = 0.010), where data were available from 206 patients.

**Figure 6:**
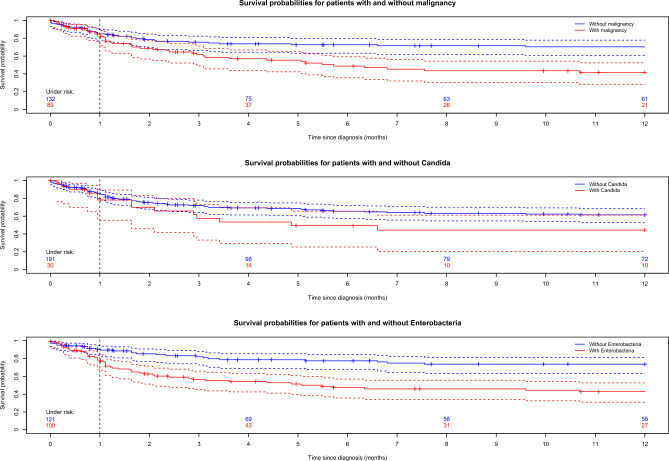
Kaplan–Meier curves along with their 95% confidence bands for patients with pyogenic liver abscesses divided with respect to underlying malignancies (top), *Candida* spp. (center) and Enterobacterales (bottom).

The Kaplan–Meier curves in Fig. 6 (top) provide estimates for all-cause mortality taking censorship into account. At 30 days, these estimates agree well with those above and one finds 15.4% in patients with malignancies compared to 15.2% in cases without malignancies. However, mortality differed after one year (58.4% malignant vs. 29.6% non-malignant) with p < 0.001 according to a log-rank test. In Fig. 6 we also present Kaplan–Meier curves for patient with and without specific pathogens namely *Candida* spp. (center) and Enterobacterales (bottom). Using a Cox model with covariates age, malignancy, *Candida* spp., Enterobacterales and *Enterococcus* spp., we obtain statistically significant hazard ratios for the underlying malignancies and Enterobacterales (see Supplementary Table S5 online).

## Discussion

This retrospective observational single-center study comprises one of the largest European data sets for PLA published to date and is – to our knowledge – the largest PLA cohort in Germany. Our data show that most PLA represent complications of biliary disorders, especially malignancies, whereas PLA caused by hematogenous spread from distant infectious foci are rare. Abdominal US, especially combined with contrast-enhanced US (CEUS), is paramount for establishing the diagnosis of PLA and deciding on further diagnostics or immediate intervention. Invasive measures (puncture, drainage) were necessary in a large proportion of cases, and the observed mortality was higher than reported before^14–16,18,34–40^.

In general, PLA represents a rare abdominal disease in Western countries^14–16,19,34^. However, with 221 cases over a period of 7 years, PLA shows a relevant incidence in our hospital (about 32 cases per year, i.e. 2–3 cases per month). This PLA frequency is comparable to previous reports from other Western hospitals^14–17,19,40–44^.

This study also confirms some well-known PLA characteristics from the literature: the preponderance of males, “typical” symptoms (e.g. abdominal pain, fever) and the predominant involvement of the right liver lobe (Table 1)^19,35–38,40,41,45,46^. Biliary malignancies, cholelithiasis, and ischemic bile duct disease were the most frequent causes for PLA in our study. A possible explanation for this observation is the interdisciplinary liver center at our hospital with a large liver transplant program; a center effect is therefore likely. On the other hand, there are indications of a general shift in etiology: whereas decades ago appendicitis, trauma, post-surgical complications or hematogenous spread were common causes, in recent years diseases of the hepatic-pancreatic-biliary system have increasingly become the cause of PLA^14,17,34–36,38,41,42,44^. This is certainly due to a changed patient clientele with older and more multi-morbid patients with underlying malignant diseases or immunosuppression, paired with an increasing willingness for medical intervention.

The frequent use of US as initial imaging method is in line with other studies^18,36,37,39^ and reflects the wide availability, time and cost-effectiveness as well as high sensitivity for identifying liver lesions^47,48^. In a retrospective study of 268 patients, Lin et al. calculated a diagnostic sensitivity for US of 86%^49^. Irrespective of the first line imaging method, 27% of patients required further imaging in our study. Special imaging procedures such as MRI or PET-CT are primarily necessary to rule out certain differential diagnoses (e.g. metastases, cysts, echinococcosis, benign liver tumors) or to characterize smaller or multiple abscesses, the exact 3D-localization, the hepatic venous anatomy or PLA-associated diseases outside the liver. In a rational diagnostic cascade, US should be used as a first-line imaging method. Depending on US results, complex and resource-intensive diagnostic procedure such as CT, CEUS or – in special cases – MRI, PET-CT or ERCP should be complemented based on individual requirements^18,22,30^.

The shift in the underlying pathogens has gained particular attention in reports on PLA^22,38,45^. In our study Enterobacterales (58%), enterococci (42%), and streptococci (18%) accounted for the vast majority of both blood and abscess cultures. In general, the observed pathogen spectrum was similar to previous PLA studies from Europe and North America^15,16,18,19,34–41,43,44,46^. It should be emphasized that hypervirulent *Klebsiella pneumoniae* was only diagnosed in one individual case within the study period^50^. The high incidence of Enterobacterales and enterococci is probably related to the high incidence of biliary diseases in this study and may also reflect the specific situation at a tertiary care hospital. The antimicrobial resistance rates of the isolated bacterial strains, in particular regarding Enterobacterales, were within a manageable range and were subject to thorough consultation and, if necessary, therapy modification by the infectious diseases consultation service and the antibiotic stewardship team. Interestingly, we observed a slight decline of general resistance rates at our institution during the study period, which can be attributed to the concurrent improvement of antibiotic stewardship measures. Initial therapy with piperacillin/tazobactam or carbapenems in particular was highly effective. The low detection rate of *Candida* spp. confirms that a routine empirical coverage of fungi seems not to be necessary, apart from severely immunosuppressed patients (e.g. neutropenia) and suspected hepatosplenic candidiasis.

Abscess or blood cultures were obtained in less than 80% of cases and joint blood and abscess cultures in only 59%. Here, we see greater potential for diagnostic improvements, e.g. through regular training of physicians on optimal specimen collection.

Despite the use of antibiotics in almost all PLA patients, the majority required additional intervention sooner or later. This was also observed in other studies^18,35–37,40,46^. In contrast to ALA, antibiotic therapy alone is usually not sufficient to treat PLA. According to Losie et al., the absence of drainage is associated with an increased 30-day mortality in PLA (univariate OR 5.8, 95% CI 1.58–21.30, p = 0.006)^34^. Regarding the optimal procedure, a recent meta-analysis concluded that catheter drainage is superior to needle aspiration in terms of treatment success and duration of antibiotic therapy, especially for large PLA (diameter > 6 cm or abscess volume > 113 mL)^25^. A German multicenter study identified a low complication rate of < 2% in 6,420 image-guided catheter drainages of the liver^51^. Therefore, indications for catheter drainage in PLA patients should be generous. In selected PLA cases (e.g. multiple or large abscesses, abscess rupture, and lack of clinical improvement after drainage and antibiotic therapy), surgery should nevertheless be performed.

The all-cause fatality rate within 30 days after PLA diagnosis was relatively high (> 14%) in our cohort compared to other studies from Western countries (range 0.3–11%)^14–16,34–40^. However, this could also be a center effect reflecting PLA patients with severe comorbidities. A higher mortality rate in PLA patients (19%) was also reported in a Spanish study published in 2007^46^. Interestingly, the outcomes in patients with and without underlying malignancies was similar within the first 30 days after PLA diagnosis in our analysis, but differed in the further course. Therefore, treatment procedures in patients with advanced malignant diseases should be reassessed if therapy attempts fail to resolve the PLA within one month.

Both positive blood cultures (OR 4.78) and detection of Enterobacterales (OR 3.55) were associated with increased 30-day mortality. This is consistent with data from other studies in which positive blood cultures in PLA patients were also associated with increased mortality^16,37^. In contrast to our results, other studies indicated an increased mortality when enterococci were detected^40,42,44^. It should be discussed whether empirical therapy should be extended to cover enterococci in selected patients with severe diseases, risk factors for enterococcal infections such as immunosuppression or previous long-term treatment with cephalosporins. Structured sampling of at least 2–3 blood culture sets has been shown to be effective in many infectious diseases, e.g., in the diagnosis of endocarditis, and similar strategies should be considered for the treatment of PLA. For appropriate microbiological diagnostics and individualized antibiotic treatment, we recommend consulting an infectious disease specialist as soon as PLA is suspected.

Limitations comprise the retrospective and monocentric study design with inherent information bias in a restricted study period of 7 years (inclusion of the years 2020 to 2022 was waived due to Covid-19-related differences in patient population). In particular, data on long-term mortality may be subject to bias. Hence, we opted not to extend the survival analyses beyond a follow-up period of one year. Our study only allows association analyses of culture-based pathogen detection with clinical conditions, but does not allow identification of causal pathways. Non-culturable pathogens elude such analysis, and early anti-infective therapy prior to specimen collection also affects the interpretation of the pathogen spectrum. In addition, laboratory methods may have changed over time.

A significant portion of the available data on PLA derives from comparable single center analysis and is, therefore, subject of respective limitations. This underscores the need for more comprehensive, prospective controlled studies to evaluate the optimal care of PLA patients in order to provide evidence-based treatment guidelines for the management of PLA.

## Conclusions

PLA is highly relevant in visceral medicine, especially in patients with biliary disorders. Abdominal ultrasound is the method of choice for diagnosis and intervention. A large proportion of patients require intervention during the course. An "aggressive" management is important for local therapy. Since positive blood cultures in general and detection of Enterobacterales determine the prognosis, careful specimen collection and appropriate empiric anti-infective treatment are crucial. In terms of survival rates, patients with malignancies do not differ from patients without malignancies within the first 30 days after diagnosis.

### Supplementary Information

Supplementary Tables.

## Data Availability

Anonymized data will be available upon request. Contact Thomas.Karlas@medizin.uni-leipzig.de.
