# Supplementary material for: Clinical management, pathogen spectrum and outcomes in patients with pyogenic liver abscess in a German tertiary-care hospital
Source: Sci Rep. 2024 Jun 5;14:12972. doi: 10.1038/s41598-024-63819-w (PMC11153614; doi:10.1038/s41598-024-63819-w)
Supplement: Supplementary file 1 — Supplementary Tables. [file 41598_2024_63819_MOESM1_ESM.docx]

Clinical management, pathogen spectrum and outcomes in patients with pyogenic liver abscess in a German tertiary-care hospital

**Supporting Information**

**Table S1.** Tabular summary of blood culture detection rates as a function of abscess culture results.

|  | Blood culture negative | Blood culture positive | Blood culture not done |
| --- | --- | --- | --- |
| Abscess culture negative | 8 | 4 | 8 |
| Abscess culture positive | 52 | 66 | 25 |
| Abscess culture not available | 19 | 24 | 15 |

**Table S2.** Overview of cultured microorganisms from blood or abscess material.

|  | **2013**  *n* (%) | **2014**  *n* (%) | **2015**  *n* (%) | **2016**  *n* (%) | **2017**  *n* (%) | **2018**  *n* (%) | **2019**  *n* (%) | **Overall**  *n* (%) |
| --- | --- | --- | --- | --- | --- | --- | --- | --- |
| *Positive culture established* | 12 (100) | 19 (100) | 24 (100) | 34 (100) | 22 (100) | 27 (100) | 33 (100) | 171 (100) |
| **Most common cultured microbial species** (n ≥5, alphabetically) | | | | | | | | |
| *Escherichia coli* | 5 (42) | 8 (42) | 5 (21) | 18 (53) | 5 (23) | 10 (37) | 10 (30) | 61 (36) |
| *Enterococcus faecium* | 2 (17) | 4 (21) | 7 (29) | 10 (29) | 6 (27) | 4 (15) | 9 (27) | 42 (25) |
| *Enterococcus faecalis* | 4 (33) | 6 (32) | 4 (17) | 6 (18) | 2 (9) | 4 (15) | 6 (18) | 32 (19) |
| *Klebsiella pneumoniae* | 1 (8) | 3 (16) | 2 (8) | 6 (18) | 7 (32) | 4 (15) | 8 (24) | 31 (18) |
| *Candida albicans* | 1 (8) | 1 (5) | 3 (12) | 9 (26) | 4 (18) | 3 (11) | 1 (3) | 22 (13) |
| *Streptococcus anginosus* group | 2 (17) | 0 (0) | 3 (12) | 4 (12) | 8 (36) | 1 (4) | 4 (12) | 22 (13) |
| *Klebsiella oxytoca* | 0 (0) | 0 (0) | 0 (0) | 2 (6) | 2 (9) | 2 (7) | 5 (15) | 11 (6) |
| *Enterobacter cloacae* complex | 1 (8) | 0 (0) | 5 (21) | 2 (6) | 0 (0) | 1 (4) | 1 (3) | 10 (6) |
| *Pseudomonas aeruginosa* | 1 (8) | 1 (5) | 1 (4) | 4 (12) | 0 (0) | 1 (4) | 2 (6) | 10 (6) |
| *Clostridium perfringens* | 0 (0) | 1 (5) | 1 (4) | 3 (9) | 0 (0) | 1 (4) | 1 (3) | 7 (4) |
| *Candida glabrata* | 0 (0) | 1 (5) | 0 (0) | 2 (6) | 1 (5) | 0 (0) | 1 (3) | 5 (3) |
| *Citrobacter freundii* complex | 1 (8) | 0 (0) | 0 (0) | 1 (3) | 1 (5) | 0 (0) | 2 (6) | 5 (3) |
| *Fusobacterium nucleatum* | 1 (8) | 0 (0) | 0 (0) | 0 (0) | 1 (5) | 1 (4) | 2 (6) | 5 (3) |
| Others^§^ | 4 (33) | 7 (37) | 15 (62) | 11 (32) | 9 (41) | 22 (81) | 17 (52) | 85 (50) |

^§^“Others” comprises species that have been detected less than 5 times during the study period (*Actinomyces israelii, Actinomyces odontolyticus, Bacteroides fragilis, Bacteroides ovatus, Bacteroides pyogenes, Bacteroides thetaiotaomicron, Bacteroides vulgatus, Campylobacter spp., Candida guilliermondii, Candida kefyr, Candida krusei, Citrobacter koseri, Dialister pneumosintes, Eikenella corrodens, Enterococcus avium, Enterococcus casseliflavus, Enterococcus hirae, Fusobacterium mortiferum, Fusobacterium necrophorum, Gemella morbillorum, Haemophilus parainfluenzae, Hafnia alvei, Klebsiella aerogenes, Lactococcus garvieae, Lactococcus lactis, Listeria monocytogenes, Micrococcus luteus, Micromonas micros, Morganella morganii, Parvimonas micra, Prevotella buccae, Prevotella intermedia, Prevotella oralis, Propionibacterium avidum, Proteus mirabilis, Rothia dentocariosa, Serratia marcescens, Staphylococcus aureus, Staphylococcus capitis, Staphylococcus epidermidis, Staphylococcus haemolyticus, Staphylococcus hominis, Staphylococcus lugdunensis, Stenotrophomonas maltophilia, Streptococcus agalactiae, Streptococcus intermedius, Streptococcus parasanguinis, Streptococcus salivarius, Streptococcus vestibularis, Veillonella parvula)*.

**Table S3.** Resistance data (2013 – 2019) of selected microorganisms at the Leipzig University Medical Center.

| **Anti-infectives** | **Year** | ***E. coli*** | | | ***K. pneumoniae*** | | | ***E. faecalis*** | | | ***E. faecium*** | | | ***C. albicans*** | | |
| --- | --- | --- | --- | --- | --- | --- | --- | --- | --- | --- | --- | --- | --- | --- | --- | --- |
|  |  | (*n*=19,364) | | | (*n*=5,710) | | | (*n*=6,480) | | | (*n*=2,977) | | | (*n*=1,174) | | |
|  |  | S | I | R | S | I | R | S | I | R | S | I | R | S | I | R |
| **Ampicillin** | 2013 | 45% | 0% | 55% | 0% | 0% | 100% |  |  |  |  |  |  |  |  |  |
|  | 2014 | 45% | 0% | 55% | 0% | 0% | 100% |  |  |  |  |  |  |  |  |  |
|  | 2015 | 43% | 3% | 54% | 0% | 0% | 100% |  |  |  |  |  |  |  |  |  |
|  | 2016* | 1% | 47% | 52% | 0% | 0% | 100% |  |  |  |  |  |  |  |  |  |
|  | 2017 | 0% | 47% | 53% | 0% | 0% | 100% |  |  |  |  |  |  |  |  |  |
|  | 2018 | 0% | 45% | 55% | 0% | 0% | 100% |  |  |  |  |  |  |  |  |  |
|  | 2019 | 1% | 48% | 51% | 0% | 1% | 99% |  |  |  |  |  |  |  |  |  |
| **Ampicillin/**  **sulbactam** | 2013 | 52% | 0% | 48% | 62% | 0% | 38% | 99% | 0% | 1% | 5% | 0% | 95% |  |  |  |
|  | 2014 | 56% | 0% | 44% | 70% | 0% | 30% | 100% | 0% | 0% | 2% | 0% | 98% |  |  |  |
|  | 2015 | 52% | 4% | 44% | 64% | 4% | 32% | 99% | 0% | 1% | 4% | 1% | 95% |  |  |  |
|  | 2016* | 2% | 55% | 43% | 0% | 67% | 33% | 99% | 0% | 1% | 6% | 0% | 94% |  |  |  |
|  | 2017 | 1% | 56% | 43% | 1% | 65% | 34% | 100% | 0% | 0% | 6% | 0% | 94% |  |  |  |
|  | 2018 | 2% | 55% | 43% | 1% | 65% | 34% | 98% | 1% | 1% | 8% | 0% | 92% |  |  |  |
|  | 2019 | 6% | 55% | 39% | 3% | 72% | 25% | 99% | 0% | 1% | 9% | 1% | 90% |  |  |  |
| **Piperacillin/ tazobactam** | 2013 | 90% | 2% | 8% | 75% | 5% | 20% |  |  |  |  |  |  |  |  |  |
|  | 2014 | 93% | 2% | 5% | 84% | 4% | 12% |  |  |  |  |  |  |  |  |  |
|  | 2015 | 93% | 2% | 5% | 83% | 6% | 11% |  |  |  |  |  |  |  |  |  |
|  | 2016 | 94% | 1% | 5% | 83% | 6% | 11% |  |  |  |  |  |  |  |  |  |
|  | 2017 | 93% | 1% | 6% | 80% | 8% | 12% |  |  |  |  |  |  |  |  |  |
|  | 2018 | 95% | 1% | 4% | 87% | 5% | 8% |  |  |  |  |  |  |  |  |  |
|  | 2019 | 95% | 1% | 4% | 87% | 6% | 7% |  |  |  |  |  |  |  |  |  |
| **Cefotaxime** | 2013 | 85% | 0% | 15% | 80% | 0% | 20% |  |  |  |  |  |  |  |  |  |
|  | 2014 | 84% | 0% | 16% | 85% | 0% | 15% |  |  |  |  |  |  |  |  |  |
|  | 2015 | 87% | 0% | 13% | 85% | 0% | 15% |  |  |  |  |  |  |  |  |  |
|  | 2016 | 86% | 0% | 14% | 85% | 0% | 15% |  |  |  |  |  |  |  |  |  |
|  | 2017 | 87% | 1% | 12% | 84% | 0% | 16% |  |  |  |  |  |  |  |  |  |
|  | 2018 | 86% | 0% | 14% | 83% | 0% | 17% |  |  |  |  |  |  |  |  |  |
|  | 2019 | 88% | 0% | 12% | 87% | 0% | 13% |  |  |  |  |  |  |  |  |  |
| **Ciprofloxacin** | 2013 | 72% | 1% | 27% | 73% | 2% | 25% |  |  |  |  |  |  |  |  |  |
|  | 2014 | 73% | 1% | 26% | 83% | 2% | 15% |  |  |  |  |  |  |  |  |  |
|  | 2015 | 78% | 1% | 21% | 82% | 3% | 15% |  |  |  |  |  |  |  |  |  |
|  | 2016 | 78% | 1% | 21% | 81% | 2% | 17% |  |  |  |  |  |  |  |  |  |
|  | 2017 | 75% | 3% | 22% | 75% | 5% | 20% |  |  |  |  |  |  |  |  |  |
|  | 2018 | 76% | 5% | 19% | 78% | 4% | 18% |  |  |  |  |  |  |  |  |  |
|  | 2019 | 83% | 3% | 14% | 86% | 4% | 10% |  |  |  |  |  |  |  |  |  |
| **Cotrimoxazole** | 2013 | 58% | 1% | 41% | 65% | 3% | 32% | 6% | 30% | 65% | 2% | 14% | 84% |  |  |  |
|  | 2014 | 65% | 1% | 33% | 76% | 1% | 23% | 0% | 48% | 52% | 1% | 27% | 72% |  |  |  |
|  | 2015 | 70% | 1% | 29% | 78% | 2% | 20% | 0% | 50% | 50% | 0% | 24% | 76% |  |  |  |
|  | 2016 | 69% | 1% | 30% | 75% | 2% | 23% | 4% | 53% | 43% | 0% | 25% | 75% |  |  |  |
|  | 2017 | 71% | 1% | 28% | 76% | 3% | 21% | 2% | 53% | 45% | 0% | 31% | 69% |  |  |  |
|  | 2018 | 74% | 1% | 25% | 78% | 2% | 20% | 2% | 75% | 23% | 0% | 38% | 62% |  |  |  |
|  | 2019 | 73% | 1% | 26% | 83% | 1% | 16% | 0% | 82% | 18% | 0% | 34% | 66% |  |  |  |
| **Imipenem** | 2013 | 100% | 0% | 0% | 97% | 0% | 3% | 99% | 0% | 1% | 4% | 0% | 96% |  |  |  |
|  | 2014 | 100% | 0% | 0% | 100% | 0% | 0% | 100% | 0% | 0% | 2% | 0% | 98% |  |  |  |
|  | 2015 | 100% | 0% | 0% | 99% | 0% | 1% | 99% | 0% | 1% | 4% | 0% | 96% |  |  |  |
|  | 2016 | 100% | 0% | 0% | 99% | 0% | 1% | 98% | 1% | 1% | 5% | 1% | 94% |  |  |  |
|  | 2017 | 100% | 0% | 0% | 100% | 0% | 0% | 99% | 0% | 1% | 5% | 1% | 94% |  |  |  |
|  | 2018 | 100% | 0% | 0% | 100% | 0% | 0% | 100% | 0% | 0% | 6% | 2% | 92% |  |  |  |
|  | 2019 | 100% | 0% | 0% | 99% | 1% | 0% | 99% | 0% | 1% | 9% | 2% | 89% |  |  |  |
| **Meropenem** | 2013 | 100% | 0% | 0% | 97% | 0% | 3% |  |  |  |  |  |  |  |  |  |
|  | 2014 | 100% | 0% | 0% | 100% | 0% | 0% |  |  |  |  |  |  |  |  |  |
|  | 2015 | 100% | 0% | 0% | 99% | 0% | 1% |  |  |  |  |  |  |  |  |  |
|  | 2016 | 100% | 0% | 0% | 99% | 0% | 1% |  |  |  |  |  |  |  |  |  |
|  | 2017 | 100% | 0% | 0% | 100% | 0% | 0% |  |  |  |  |  |  |  |  |  |
|  | 2018 | 100% | 0% | 0% | 100% | 0% | 0% |  |  |  |  |  |  |  |  |  |
|  | 2019 | 100% | 0% | 0% | 100% | 0% | 0% |  |  |  |  |  |  |  |  |  |
| **Vancomycin** | 2013 |  |  |  |  |  |  | 99% | 0% | 1% | 57% | 0% | 43% |  |  |  |
|  | 2014 |  |  |  |  |  |  | 99% | 0% | 1% | 67% | 0% | 33% |  |  |  |
|  | 2015 |  |  |  |  |  |  | 99% | 0% | 1% | 61% | 0% | 39% |  |  |  |
|  | 2016 |  |  |  |  |  |  | 99% | 0% | 1% | 60% | 0% | 40% |  |  |  |
|  | 2017 |  |  |  |  |  |  | 99% | 0% | 1% | 59% | 0% | 41% |  |  |  |
|  | 2018 |  |  |  |  |  |  | 100% | 0% | 0% | 56% | 0% | 44% |  |  |  |
|  | 2019 |  |  |  |  |  |  | 100% | 0% | 0% | 71% | 0% | 29% |  |  |  |
| **Linezolid** | 2013 |  |  |  |  |  |  | 99% | 0% | 1% | 100% | 0% | 0% |  |  |  |
|  | 2014 |  |  |  |  |  |  | 100% | 0% | 0% | 100% | 0% | 0% |  |  |  |
|  | 2015 |  |  |  |  |  |  | 100% | 0% | 0% | 100% | 0% | 0% |  |  |  |
|  | 2016 |  |  |  |  |  |  | 100% | 0% | 0% | 100% | 0% | 0% |  |  |  |
|  | 2017 |  |  |  |  |  |  | 100% | 0% | 0% | 100% | 0% | 0% |  |  |  |
|  | 2018 |  |  |  |  |  |  | 100% | 0% | 0% | 100% | 0% | 0% |  |  |  |
|  | 2019 |  |  |  |  |  |  | 100% | 0% | 0% | 99% | 0% | 1% |  |  |  |
| **Tigecycline** | 2013 | *NA* | *NA* | *NA* | *NA* | *NA* | *NA* | 80% | 0% | 20% | 96% | 0% | 4% |  |  |  |
|  | 2014 | *NA* | *NA* | *NA* | *NA* | *NA* | *NA* | 100% | 0% | 0% | 92% | 3% | 5% |  |  |  |
|  | 2015 | 100% | 0% | 0% | *NA* | *NA* | *NA* | 100% | 0% | 0% | 96% | 1% | 3% |  |  |  |
|  | 2016 | 100% | 0% | 0% | 90% | 4% | 6% | 93% | 0% | 7% | 96% | 1% | 3% |  |  |  |
|  | 2017 | 100% | 0% | 0% | 90% | 6% | 4% | 95% | 3% | 2% | 96% | 2% | 2% |  |  |  |
|  | 2018 | 99% | 1% | 0% | 93% | 4% | 3% | 98% | 1% | 1% | 99% | 0% | 1% |  |  |  |
|  | 2019 | 97% | 2% | 1% | 92% | 4% | 4% | 98% | 0% | 2% | 98% | 0% | 2% |  |  |  |
| **Fluconazole** | 2013 |  |  |  |  |  |  |  |  |  |  |  |  | 98% | 1% | 1% |
|  | 2014 |  |  |  |  |  |  |  |  |  |  |  |  | 99% | 0% | 1% |
|  | 2015 |  |  |  |  |  |  |  |  |  |  |  |  | 100% | 0% | 0% |
|  | 2016 |  |  |  |  |  |  |  |  |  |  |  |  | 100% | 0% | 0% |
|  | 2017 |  |  |  |  |  |  |  |  |  |  |  |  | 100% | 0% | 0% |
|  | 2018 |  |  |  |  |  |  |  |  |  |  |  |  | 100% | 0% | 0% |
|  | 2019 |  |  |  |  |  |  |  |  |  |  |  |  | 100% | 0% | 0% |
| **Caspofungin** | 2013 |  |  |  |  |  |  |  |  |  |  |  |  | 100% | 0% | 0% |
|  | 2014 |  |  |  |  |  |  |  |  |  |  |  |  | 100% | 0% | 0% |
|  | 2015 |  |  |  |  |  |  |  |  |  |  |  |  | 100% | 0% | 0% |
|  | 2016 |  |  |  |  |  |  |  |  |  |  |  |  | 100% | 0% | 0% |
|  | 2017 |  |  |  |  |  |  |  |  |  |  |  |  | 100% | 0% | 0% |
|  | 2018 |  |  |  |  |  |  |  |  |  |  |  |  | 100% | 0% | 0% |
|  | 2019 |  |  |  |  |  |  |  |  |  |  |  |  | 100% | 0% | 0% |

Resistance assessments were carried out in accordance with the standards of *European Committee on Antimicrobial Susceptibility Testing* (EUCAST, www.eucast.org) valid at the respective year. *Change to the EUCAST breakpoint this year. *NA*: Not available *or* not enough resistance data available (*n* < 10). Shaded area: No resistance testing performed and no EUCAST breakpoints available respectively.

**Table S4.** Overview of the frequencies of individual anti-infectives used for the treatment of pyogenic liver abscesses (sorted by descending frequency).

| **Anti-infective agent** | **2013** | **2014** | **2015** | **2016** | **2017** | **2018** | **2019** | **Overall** |
| --- | --- | --- | --- | --- | --- | --- | --- | --- |
| Piperacillin/tazobactam | 6 | 11 | 17 | 15 | 11 | 17 | 15 | 92 |
| Imipenem | 9 | 9 | 10 | 14 | 13 | 7 | 17 | 79 |
| Metronidazole | 7 | 8 | 7 | 14 | 11 | 10 | 13 | 70 |
| Cefotaxime | 1 | 4 | 4 | 6 | 9 | 10 | 15 | 49 |
| Ciprofloxacin | 9 | 7 | 5 | 12 | 2 | 4 | 8 | 47 |
| Ceftriaxone | 5 | 6 | 3 | 9 | 3 | 4 | 0 | 30 |
| Linezolid | 1 | 1 | 6 | 5 | 6 | 4 | 7 | 30 |
| Vancomycin | 0 | 5 | 9 | 1 | 2 | 5 | 6 | 28 |
| Ampicillin/sulbactam | 1 | 4 | 6 | 3 | 1 | 2 | 2 | 19 |
| Meropenem | 0 | 0 | 3 | 2 | 2 | 8 | 2 | 17 |
| Caspofungin | 1 | 1 | 3 | 3 | 4 | 1 | 1 | 14 |
| Tigecycline | 0 | 0 | 3 | 4 | 1 | 1 | 4 | 13 |
| Cefuroxime | 1 | 2 | 2 | 2 | 2 | 0 | 1 | 10 |
| Fluconazole | 0 | 1 | 1 | 3 | 1 | 1 | 0 | 7 |
| Levofloxacin | 1 | 1 | 0 | 1 | 0 | 2 | 1 | 6 |
| Clindamycin | 0 | 0 | 1 | 1 | 0 | 2 | 2 | 6 |
| Ceftazidime | 0 | 0 | 1 | 2 | 0 | 1 | 1 | 5 |
| Others^†^ | 2 | 1 | 5 | 2 | 6 | 6 | 5 | 27 |

^†^“Others” comprises anti-infectives that have been used less than five times during the study period (gentamicin *n*=3, moxifloxacin *n*=3, penicillin *n*=3, voriconazole *n*=3, cefpodoxime *n*=2, tobramycin *n*=2, amikacin *n*=1, amoxicillin *n*=1, ampicillin *n*=1, anidulafungin *n*=1, micafungin *n*=1, erythromycin *n*=1, flucloxacillin *n*=1, rifampicin *n*=1).

**Table S5.** Result summary from a Cox model with covariates: age, malignancy, *Candida* spp., Enterobacterales, and enterococci.

|  | **Hazard ratio** | **95% CI** | **P value** |
| --- | --- | --- | --- |
| **Age (per 10 years)** | 1.09 | 0.90 to 1.30 | 0.37 |
| **Malignancy** | 1.90 | 1.19 to 3.02 | **0.0073** |
| ***Candida* spp.** | 1.63 | 0.89 to 3.00 | 0.11 |
| **Enterobacterales** | 2.41 | 1.48 to 3.92 | **<0.001** |
| ***Enterococcus* spp.** | 1.39 | 0.86 to 2.26 | 0.18 |
